# Involvement of OsGF14b Adaptation in the Drought Resistance of Rice Plants

**DOI:** 10.1186/s12284-019-0346-2

**Published:** 2019-11-14

**Authors:** Jianping Liu, Xinjiao Sun, Wencheng Liao, Jianhua Zhang, Jiansheng Liang, Weifeng Xu

**Affiliations:** 1 Center for Plant Water-use and Nutrition Regulation and College of Life Sciences, Joint International Research Laboratory of Water and Nutrient in Crop, Fujian Agriculture and Forestry University, Jinshan, Fuzhou, 350002 China; 2 Department of Biology, Hong Kong Baptist University, Hong Kong, China; 3 Department of Biology, Southern University of Science and Technology, Shenzhen, 518055 China

**Keywords:** ABA, Rice, 14–3-3, Drought resistance, OsGF14b

## Abstract

**Background:**

Drought stress is one of the major abiotic stresses that restrict plant growth and development. 14–3-3 proteins have been validated to regulate many biological processes in plants. Previous research demonstrated that OsGF14b plays different roles in panicle and leaf blast resistance. In this study, we researched the function of OsGF14b in drought resistance in rice.

**Findings:**

Here, we report that *OsGF14b* was strongly induced by soil drought stress. In comparison with wild type (WT), the *osgf14b* mutant exhibited improved resistance to drought and osmotic stress by changing the content of stress-relevant parameters, complementation of the *osgf14b* mutant restored the drought sensitivity to WT levels, whereas the *OsGF14b*-overexpression lines exhibited enhanced sensitivity to drought and osmotic stress. The *osgf14b* mutant plants were hypersensitive to abscisic acid (ABA), while the *OsGF14b*-overexpression plants showed reduced sensitivity to ABA. Furthermore, mutation and overexpression of *OsGF14b* affected the expression of stress-related genes under normal growth conditions and/or drought stress conditions.

**Conclusions:**

We have demonstrated that OsGF14b is involved in the drought resistance of rice plants, partially in an ABA-dependent manner.

## Findings

Drought is one of the main abiotic stresses affecting plant growth and yield. Sessile plants have evolved various effective mechanisms to cope with drought stress (Hu and Xiong 2014). Obtaining a better understanding of the molecular and genetic mechanism by which plants respond to drought stress has been the subject of intensive research over the past decade, and is expected to provide and essential foundation for future breeding and genetic engineering strategies (Xiang et al. 2008; Marshall et al. 2012; Tang et al. 2016; Srivastava et al. 2017; Liang et al. 2018; Lee et al. 2018; Yao et al., 2018).

14–3-3 proteins mainly function through binding and modulating the function of phosphorylated client proteins (de Boer et al. 2013). These are localized to various subcellular compartments and regulate a wide range of cellular processes (Paul et al. 2012). In higher plants, 14–3-3 proteins comprise a protein family and play important roles in regulating plant development and stress responses (Comparot et al. 2003; Denison et al. 2011). Some studies have implicated the function of 14–3-3 s in drought resistance from *Arabidopsis*, maize and *Glycine soja* (He et al. 2015; Campo et al. 2012; Sun et al. 2014). In rice, at least eight 14–3-3 isoforms (OsGF14 a-h) have been identified, and these isoforms display different expression patterns under various biotic and abiotic stresses (Chen et al. 2006; Xu and Shi 2006; Yashvardhini et al. 2018). The different roles of *OsGF14e* and *OsGF14b* in disease resistance have been reported (Manosalva et al. 2011; Liu et al. 2016b; Liu et al. 2016a). However, only *OsGF14c*’s roles in drought resistance were confirmed (Ho et al. 2013), and the functions of the other rice 14–3-3 proteins in this process are still unknown.

**Fig. 1:**
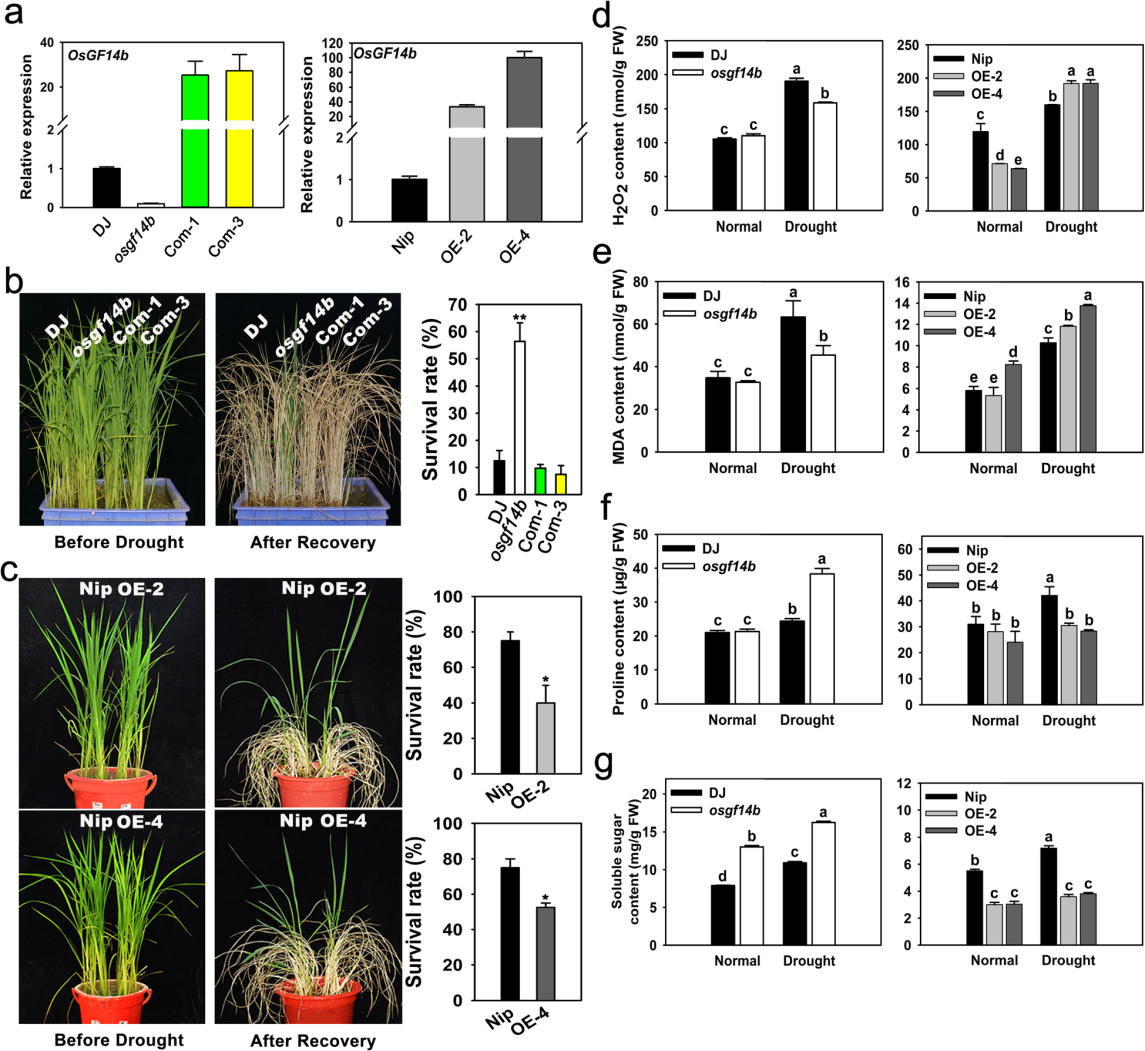
Phenotypes of the *osgf14b* mutant, complementation and *OsGF14b*-OE lines under drought stress treatment at the seedling stage. **a** Expression analysis of *OsGF14b* in the *osgf14b* mutant, complementation and *OsGF14b*-OE lines. The rice *Actin1* gene was used as the internal control. Error bars represent the SE of three biological replicates. **b** The *osgf14b* mutant showed increased drought resistance. The 5.5- to 6.5-leaf stage seedlings of DJ, *osgf14b* and complementation lines (about 20 seedlings for each genotype) were subjected to drought stress without water for 12 d and then recovered for 7 d. The seedlings with newly growing leaf blades were counted as surviving plants and the survival rates were recorded. Error bars represent the SE of three biological replicates (**, *P* < 0.01, by Student’s *t*-test). **c** The *OsGF14b*-OE lines were more sensitive to drought stress treatment. The 5.5- to 6.5-leaf stage seedlings of Nip and *OsGF14b*-OE lines (about 10 seedlings for each genotype) were subjected to drought stress without water for 8 d and then recovered for 7 d. The seedlings with newly growing leaf blades were counted as surviving plants and the survival rates were recorded. Error bars represent the SE of three replicates (*, *P* < 0.05, by Student’s *t*-test). **d-g** The H_2_O_2_, MDA, proline and soluble sugar content in the WT and transgenic plants (mutant and OE) under normal growth and drought stress conditions. Error bars represent the SE of three biological replicates. Statistical differences are labeled with different letters according to the LSD test (*P* < 0.05, one-way ANOVA)

Chen et al. (2006) reported that *OsGF14b* was rapidly induced at 2–4 h by the PEG6000 (drought-mimic). To further confirm this, we applied quantitative real-time PCR (qRT-PCR) to examine the expression of *OsGF14b* under prolonged soil drought treatment (withholding water) at the 4-leaf stage. It was found that *OsGF14b* was strongly induced at 1 d (2.3-fold) and 2 d (3-fold), and then the transcripts returned to the pre-treatment level at 3 d and 4 d, which finally remained at a higher level (2.3-fold) after recovery (Additional file 1: Figure S1). According to the above results, we speculated that OsGF14b may play a positive role in regulating drought resistance. We firstly searched for RISD (Rice T-DNA Insertion Sequence Database) and purchased the heterozygous mutant 2D-00086, a transfer DNA (T-DNA) insertion line in the japonica rice DongJin (DJ) background. The T-DNA was inserted into the promoter of *OsGF14b*, 745 bp upstream of the translation initiation site (ATG), and the homozygous mutant named *osgf14b* was identified by PCR analysis (Additional file 1: Figure S2 and Additional file 2: Table S1). The expression level of *OsGF14b* in the mutant was dramatically repressed (Fig. 1a). For drought stress, the *osgf14b* mutant and DJ seedlings (5.5- to 6.5-leaf stage) were transplanted to plastic basins with a mixture of sand and soil (1:1), and then the irrigation was withheld for 12 d, followed by re-watering for 7 d (Additional file 3). Contrary to our prediction, the *osgf14b* mutant showed increased resistance to drought stress. After recovery, about 56.3% of the *osgf14b* mutant seedlings survived compared with 12.5% of DJ seedlings (Fig. 1b). Importantly, complementation of the *osgf14b* mutant by introducing *OsGF14b* coding sequence under control of 35S promoter restored the drought sensitivity to DJ levels, thereby providing compelling proof that loss of *OsGF14b* function is causative for the observed drought-resistant mutant phenotype (Fig. 1a, b). To further validate the function of OsGF14b in drought stress response, we requested and obtained two independent *OsGF14b*-overexpression (OE) lines (OE-2 and OE-4) based on the background of Nipponbare (Nip), in which the *OsGF14b* gene was driven by 35S promoter and its expression level was significantly increased (Fig. 1a). Under normal growth conditions, we did not observe any phenotypic differences between these two overexpression lines and Nip. Under the drought stress treatment (without water for 8 d and recovered for 7 d), both OE-2 and OE-4 became more sensitive than Nip (Fig. 1c). After recovery, the survival rates of the *OsGF14b*-OE lines (40.0%–52.5%) were significantly lower than that of Nip (75%). Together, these observations supported that OsGF14b may act as a negative regulator in drought resistance.

Stomatal status is generally important for drought response in plants, so we measured the stomatal conductance of the WT and transgenic plants (mutant and OE) under normal and drought conditions at 5.5- to 6.5-leaf stage. Under normal conditions, the stomatal conductance of *osgf14b* was significantly higher than that of DJ, whereas the stomatal conductance of *OsGF14b*-OE lines was significantly lower than that of Nip; Under drought conditions (without water for 3 d), the stomatal conductance of all plants was decreased compared with under normal conditions, but there was no significant difference between the WT and transgenic plants (Additional file 1: Figure S3). The results showed that although OsGF14b could negatively regulate the stomatal conductance under normal conditions, but had almost no impact on that under drought conditions, and stomatal conductance may be not associated with the drought resistance negatively regulated by OsGF14b.

Stresses usually cause damage in plants via oxidative stress involving the generation of reactive oxygen species (ROS), such as hydrogen peroxide (H_2_O_2_) (Zhu 2001). Malondialdehyde (MDA) is an indicator of oxidative attack on membrane lipids and ion leakage reflects membrane injury (Ouyang et al. 2010). Thus, we tested the H_2_O_2_ and MDA content of the leaves from transgenic (mutant or OE) and WT plants (DJ or Nip). After drought stress, the H_2_O_2_ and MDA content in the *osgf14b* mutant were all less than DJ, whereas the *OsGF14b*-OE lines accumulated more H_2_O_2_ and MDA than Nip. Under normal conditions, we found that these two *OsGF14b*-OE lines had fewer H_2_O_2_ than Nip, and OE-4 had higher MDA than Nip, but there were no significant differences on these between *osgf14b* and DJ (Fig.1d, e). Proline and soluble sugar are two important solutes in plant cells for improving drought resistance by increasing osmotic pressure (Zhou et al. 2009). Furthermore, we also examined the content of proline and soluble sugar. After drought stress, compared with WT, the proline levels of the *osgf14b* mutant increased, but those of *OsGF14b-OE lines* declined. All tested plants had similar proline content under normal growth conditions (Fig. 1f). When the soluble sugar content was compared, the *osgf14b* mutant showed higher soluble sugar levels than DJ, while the *OsGF14b*-OE lines showed lower sugar levels than Nip under both normal and drought conditions (Fig. 1g). Taken together, these results suggested that OsGF14b may negatively regulate the resistance to drought stress via changing the content of stress-relevant parameters.

**Fig. 2:**
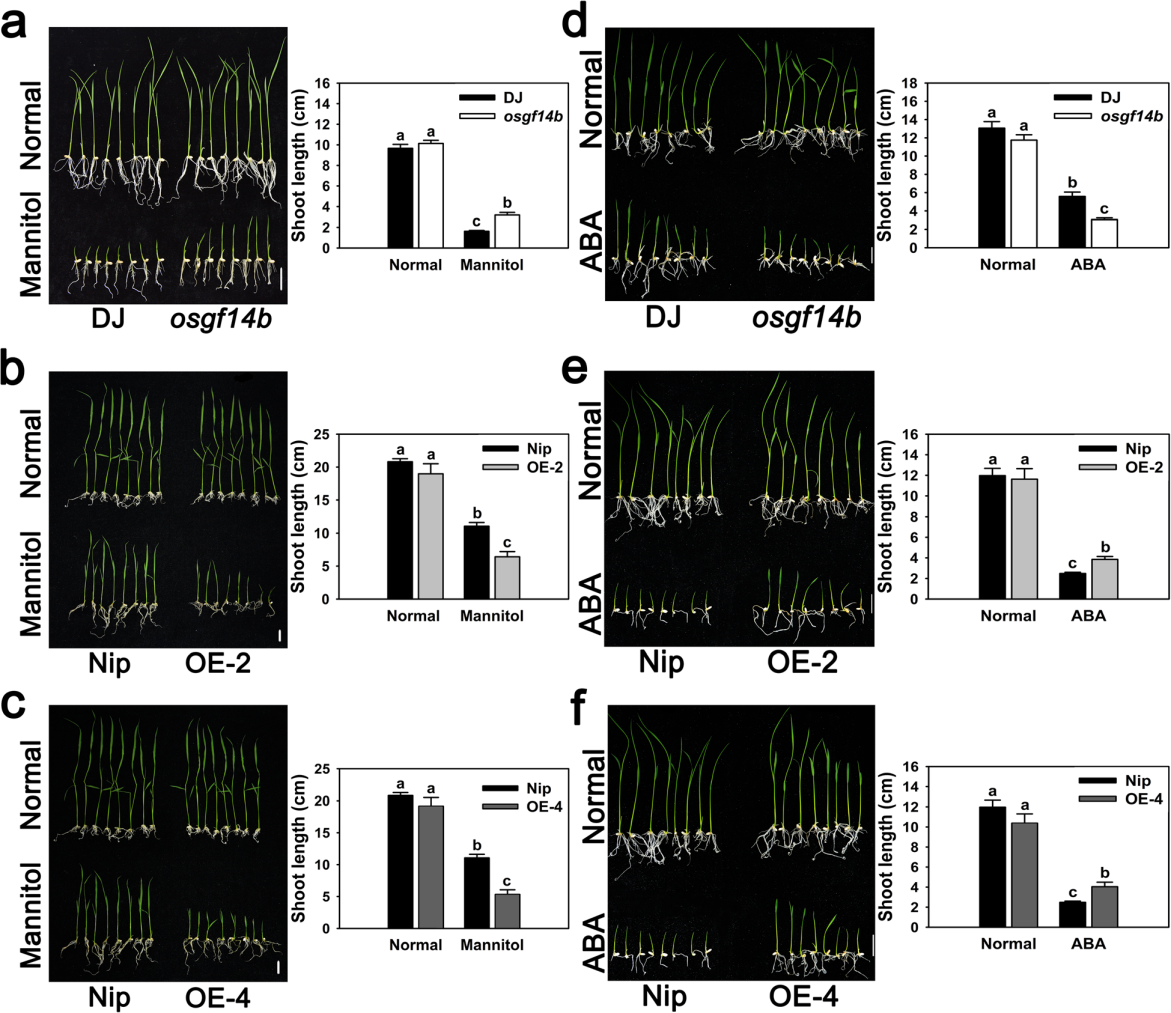
Osmotic resistance and ABA sensitivity assay of the *osgf14b* mutant and the *OsGF14b*-OE lines. **a-c** The mutant and OE lines were treated with 200 mM mannitol, under normal conditions (no addition of mannitol) as control. **d-f** The mutant and OE lines were treated with 5 μM ABA, under normal conditions (no addition of ABA) as control. Shoot length was measured to estimate the resistance and sensitivity of the WT, *osgf14b* and *OsGF14b*-OE lines. Error bars represent the SE of three biological replicates. Statistical differences are labeled with different letters according to the LSD test (*P* < 0.05, one-way ANOVA)

In order to investigate if OsGF14b functions under osmotic stress, we first sowed the surface-sterilized seeds on normal 1/2 MS medium and 10% PEG4000 supplemented-1/2 MS medium. During the germination, we calculated the germination rate at different times (1 d, 2 d, 3 d, 4 d, 5 d, 6 d and 7 d). Under normal conditions, there was no difference on the germination rate among all the genotypes, and all the genotypes started to germinate at 2 d; under the conditions of PEG4000, all the genotypes started to germinate at 3 d. From 3 d to 5 d, the germination rate of *osgf14b* was always higher than DJ, but the germination rate of *OsGF14b*-OE lines was always lower than Nip. Notably, the degree of difference on germination rate between *osgf14b* and DJ was obviously higher than that between *OsGF14b*-OE lines and Nip (Additional file 1: Figure S4). Accordingly, at germination level, the drought response in the mutant may be earlier and greater, and the drought response in *OsGF14b*-OE lines may be later and weaker. In addition, after germination on normal 1/2 MS medium, the transgenic plants together with WT control were subjected to 200 mM mannitol treatment. When grown in normal medium, there were no obvious distinctions on shoot length between the transgenic and WT plants. When grown in mannitol-supplemented medium, the *osgf14b* mutant had significantly longer shoot than DJ, whereas the *OsGF14b*-OE lines had shorter shoot than Nip (Fig. 2a, b, c). The results demonstrated that OsGF14b also play a negative role in osmotic resistance, which was consistent with its role in drought resistance.

Abscisic acid (ABA) signaling plays major roles in the drought stress (Zhang et al. 2006; Tang et al. 2016), and two previous studies showed that *OsGF14b* could be strongly induced by ABA (Chen et al. 2006; Yao et al. 2007). So we tested if OsGF14b is involved in ABA sensitivity of rice, which is an important aspect of ABA-dependent regulation. The *osgf14b* mutant and two OE lines (OE-2 and OE-4) were treated with 5 μM ABA, together with WT control. As shown in Fig. 2d, the *osgf14b* mutant seedlings were more sensitive to ABA compared to DJ. Moreover, the shoot length of the *osgf14b* mutant was significantly shorter than that of DJ under ABA treatment, but there was no significant difference under normal conditions. On the contrary, we found that the ABA sensitivity of *OsGF14b*-OE seedlings was decreased compared to Nip. In addition, the shoot length of *OsGF14b*-OE lines was much longer than that of Nip under ABA treatment. Nevertheless, no significant difference in these phenotypes was observed under normal conditions (Fig. 2e, f). Taken together, these results indicated that OsGF14b functions as a negative regulator of ABA signaling.

**Fig. 3:**
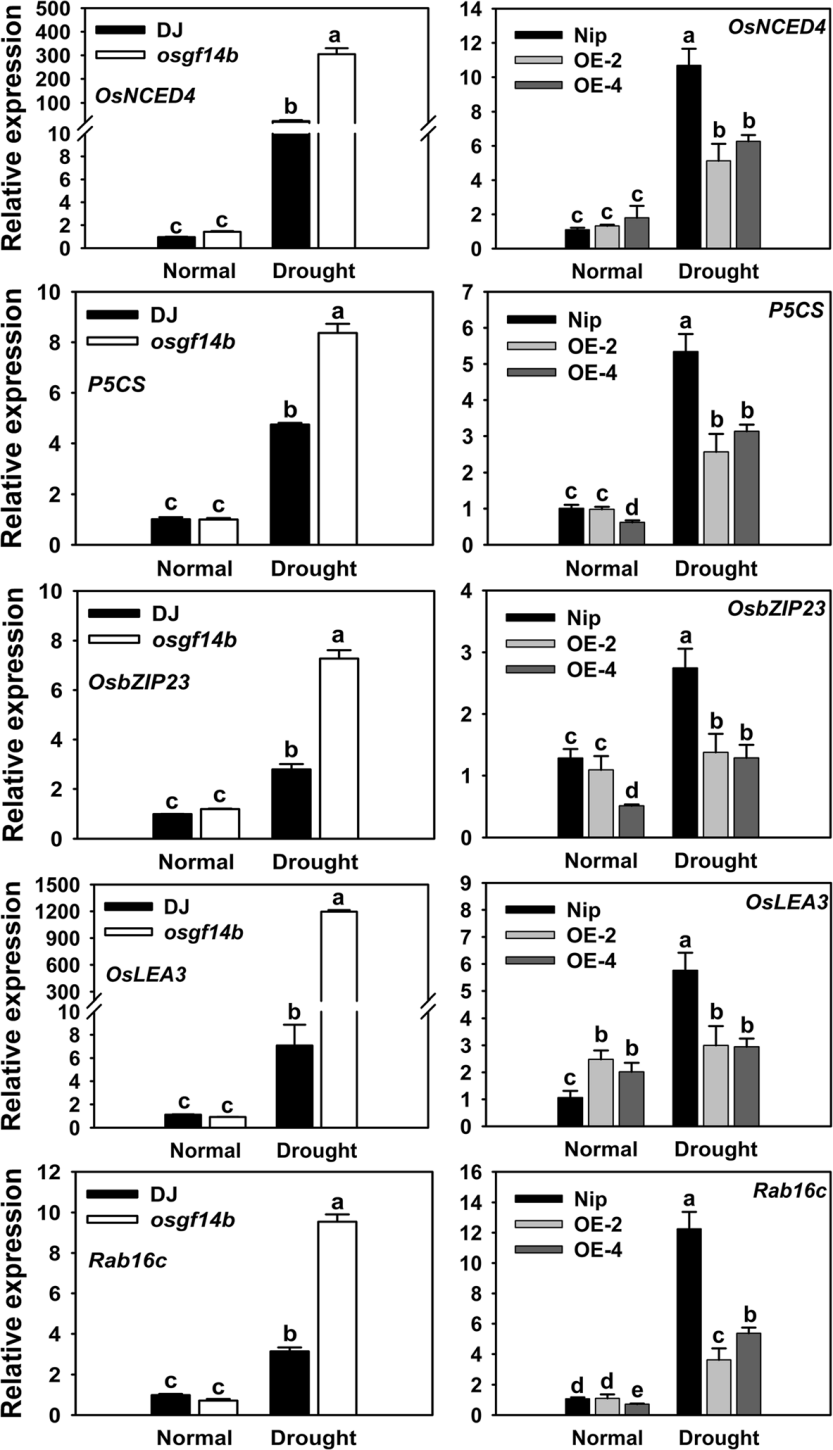
The expression of abiotic stress-responsive genes in the WT and transgenic plants (mutant and OE) under normal growth and drought stress conditions. The rice *Actin1* gene was used as the internal control. Error bars represent the SE of three biological replicates. Statistical differences are labeled with different letters according to the LSD test (*P* < 0.05, one-way ANOVA)

To explore the possible molecular mechanisms by which OsGF14b negatively regulates drought resistance in rice, we determined the expression profiles for several well-known stress-responsive genes under normal growth and drought conditions. These included *OsNCED4*, encoding protein involved in ABA biosynthesis (Zhu et al. 2009); *P5CS*, encoding a rate-limiting enzyme involved the biosynthesis of proline (Hien et al. 2003); *OsbZIP23*, encoding a typical stress-related bZIP-type transcription factor (Xiang et al. 2008); *OsLEA3* and *Rab16c*, encoding late embryogenesis abundant (LEA) proteins (Xiao et al. 2007; El-Esawi and Alayafi 2019). Our results displayed that compared to under normal conditions, the expression of *OsNCED4*, *P5CS*, *OsbZIP23*, *OsLEA3* and *Rab16c* was constitutively elevated in the transgenic (mutant and OE) and WT plants under drought stress conditions. However, after drought stress, the expression levels of these genes were significantly higher in the *osgf14b* mutant than that in DJ, and lower expression levels of these genes in the *OsGF14b*-OE lines were observed compared with Nip (Fig. 3). These results showed that OsGF14b may negatively regulate drought resistance by altering the expression of stress-responsive genes.

In conclusion, in this study we have demonstrated that OsGF14b is involved in the rice drought and osmotic resistance via changing the contents of stress-relevant parameters and the expression of stress-related genes, partially in an ABA-dependent manner. This findings presented here will provide a novel insight into the function of OsGF14b in rice.

## Additional Files

**Additional file 1. Figure S1.** Expression levels of *OsGF14b* under soil drought stress treatment. **Figure S2.** Schematic diagram of the *OsGF14b* gene and PCR-based genotyping for the *osgf14b* homozygous mutant. **Figure S3.** Stomatal conductance of the WT and transgenic plants under normal and drought conditions. **Figure S4.** Seeds germination rate of the WT and transgenic lines on normal medium and 10% PEG4000 supplemented-medium.

**Additional file 2: Table S1.** List of primers used in this study (F, forward primer; R, reverse primer; q, quantitative RT-PCR).

**Additional file 3.** Materials and methods.

## Data Availability

All data supporting the conclusions of this article are provided within the article (and its additional files).

## Abbreviations

ABA: Abscisic acid
CDS: Coding sequence
d: day
DJ: DongJin
H_2_O_2_: Hydrogen peroxide
LSD: Least significant difference
MDA: Malondialdehyde
MS: Murashige and skoog
Nip: Nipponbare
OE: Overexpression
PEG: Polyethylene glycol
qRT-PCR: Quantitative real time polymerase chain reaction
RISD DB: Rice T-DNA Insertion Sequence Database
ROS: Reactive oxygen species
WT: Wild type
