# Supplementary material for: Involvement of OsGF14b Adaptation in the Drought Resistance of Rice Plants
Source: Rice (N Y). 2019 Nov 14;12:82. doi: 10.1186/s12284-019-0346-2 (PMC6856252; doi:10.1186/s12284-019-0346-2)
Supplement: Supplementary file 1 — Additional file 1. Figure S1. Expression levels of OsGF14b under soil drought stress treatment. Figure S2. Schematic diagram of the OsGF14b gene and PCR-based genotyping for the osgf14b homozygous mutant. Figure S3. Stomatal conductance of the WT and transgenic plants under normal and drought conditions. Figure S4. Seeds germination rate of the WT and transgenic lines on normal medium and 10% PEG4000 supplemented-medium. [file 12284_2019_346_MOESM1_ESM.pptx]

## Slide 1
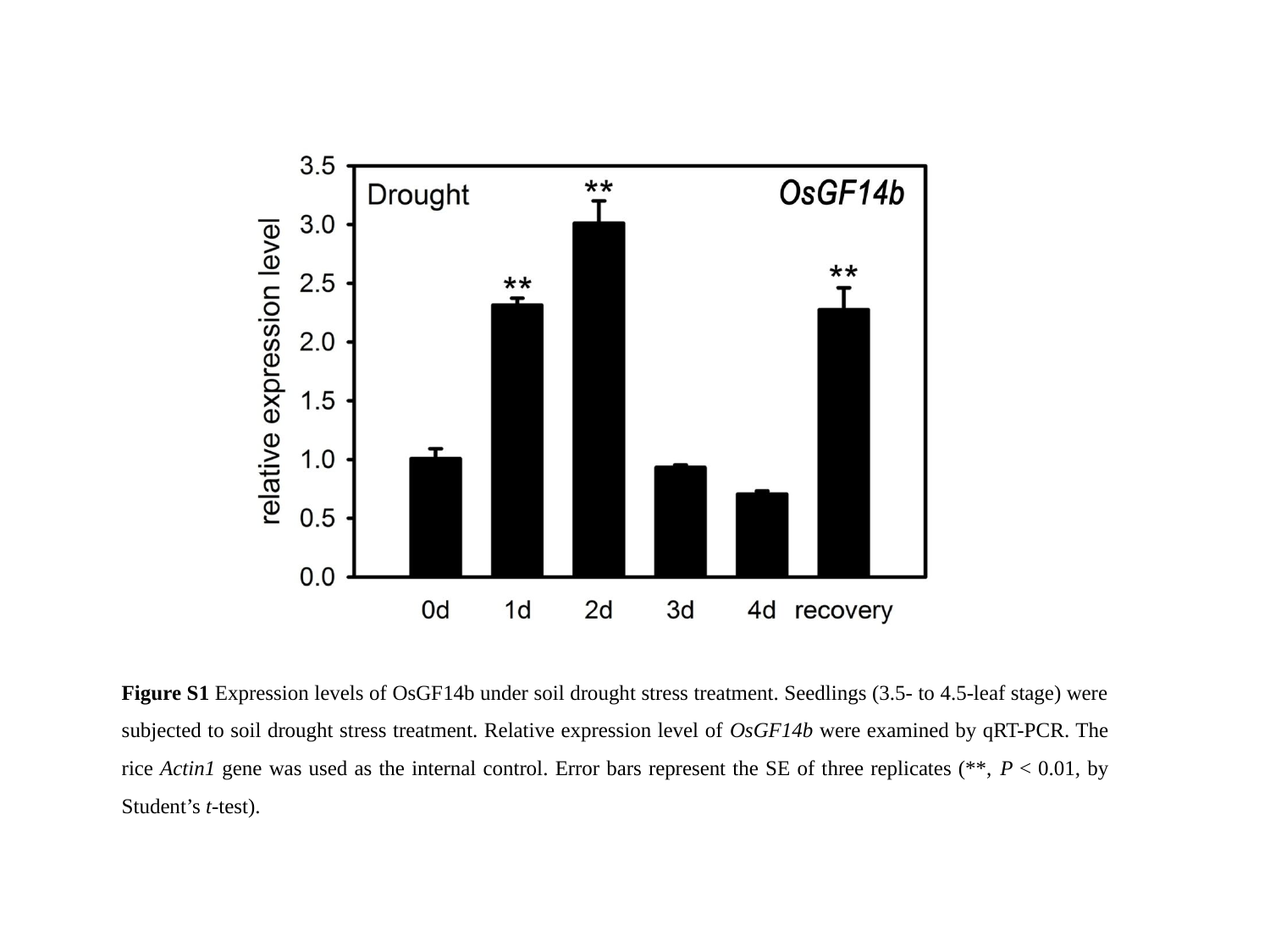

Figure S1 Expression levels of OsGF14b under soil drought stress treatment. Seedlings (3.5- to 4.5-leaf stage) were subjected to soil drought stress treatment. Relative expression level of OsGF14b were examined by qRT-PCR. The rice Actin1 gene was used as the internal control. Error bars represent the SE of three replicates (**, P < 0.01, by Student’s t-test).

## Slide 2
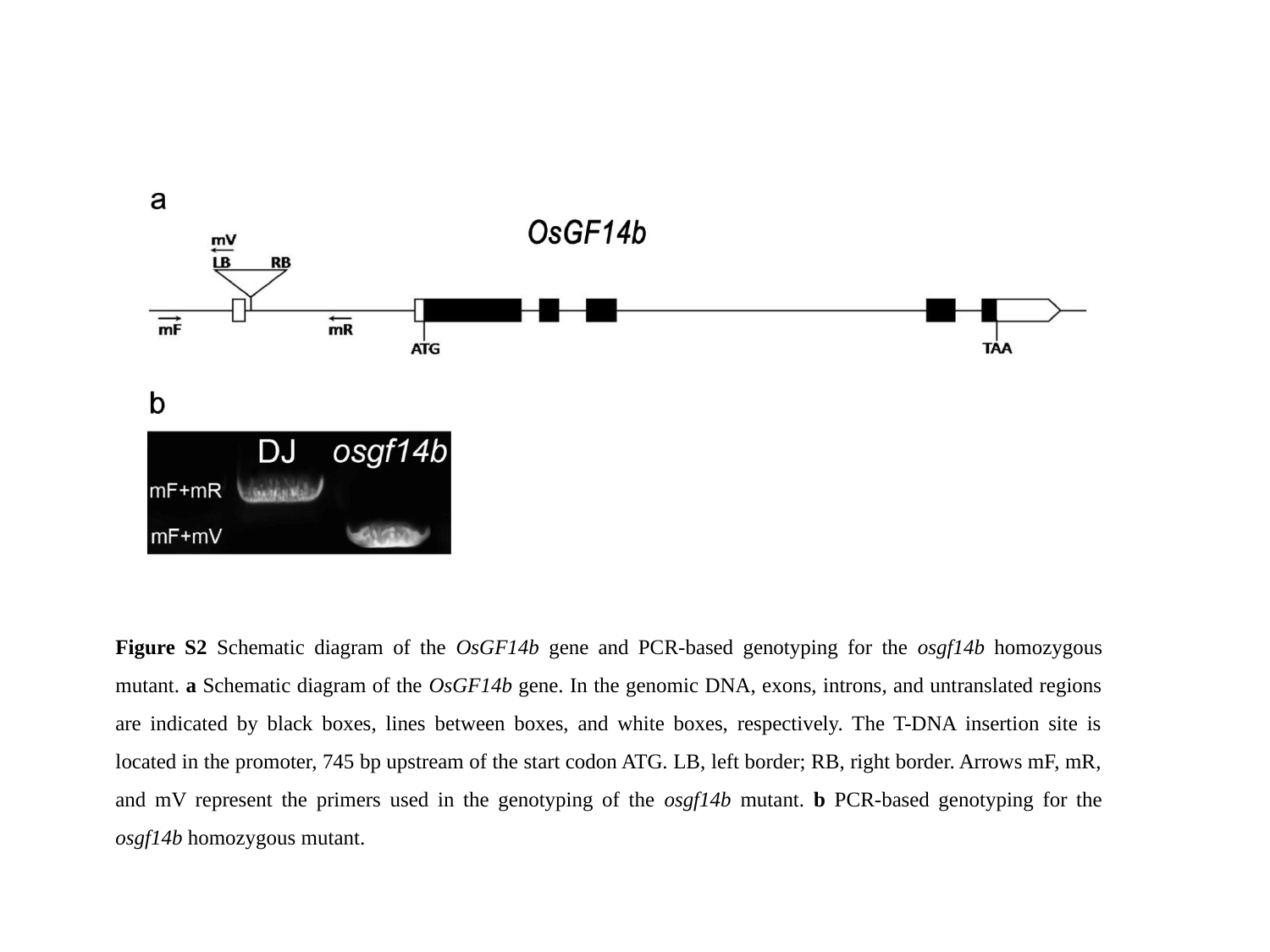

Figure S2 Schematic diagram of the OsGF14b gene and PCR-based genotyping for the osgf14b homozygous mutant. a Schematic diagram of the OsGF14b gene. In the genomic DNA, exons, introns, and untranslated regions are indicated by black boxes, lines between boxes, and white boxes, respectively. The T-DNA insertion site is located in the promoter, 745 bp upstream of the start codon ATG. LB, left border; RB, right border. Arrows mF, mR, and mV represent the primers used in the genotyping of the osgf14b mutant. b PCR-based genotyping for the osgf14b homozygous mutant.

## Slide 3
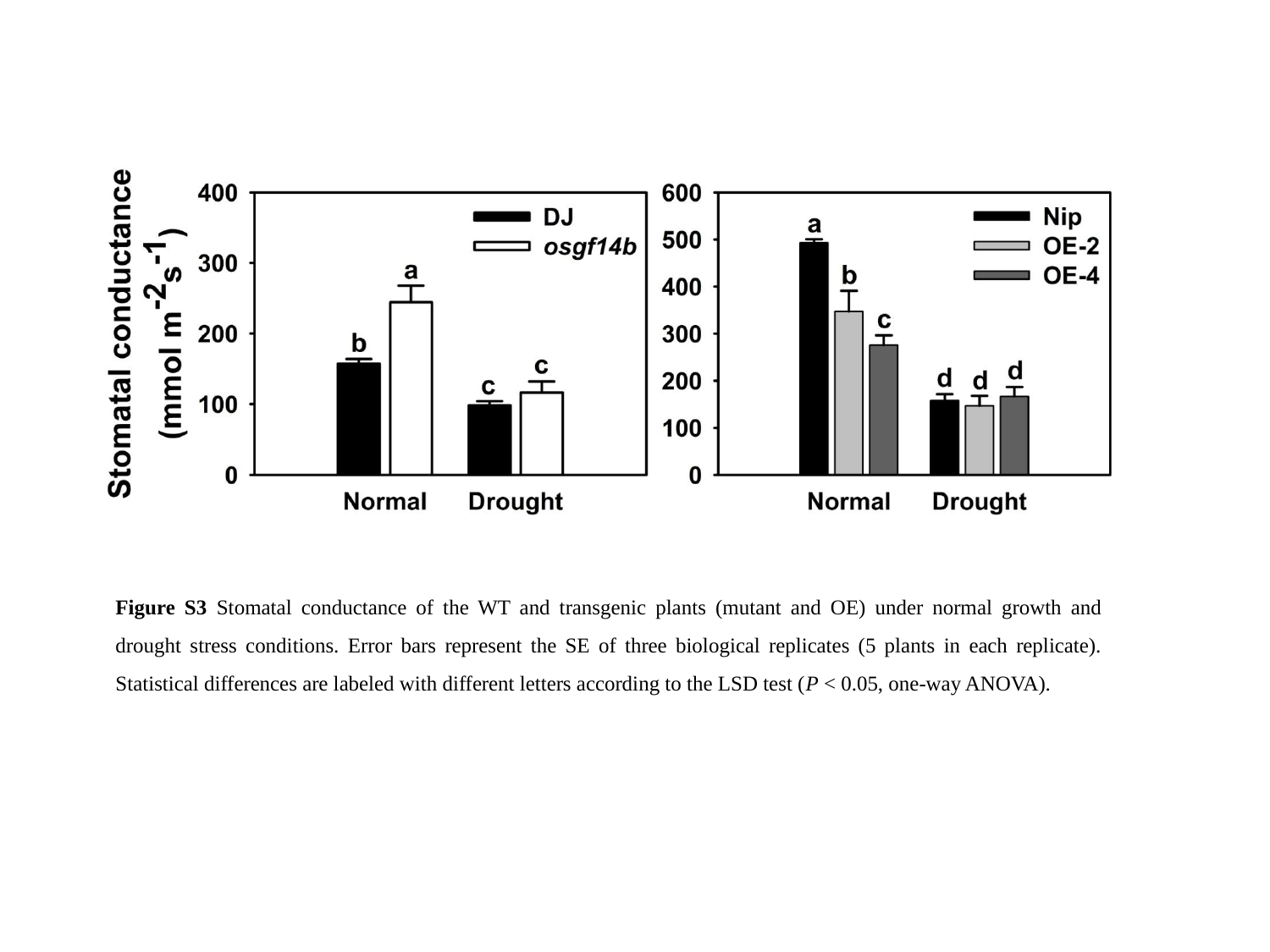

Figure S3 Stomatal conductance of the WT and transgenic plants (mutant and OE) under normal growth and drought stress conditions. Error bars represent the SE of three biological replicates (5 plants in each replicate). Statistical differences are labeled with different letters according to the LSD test (P < 0.05, one-way ANOVA).

## Slide 4
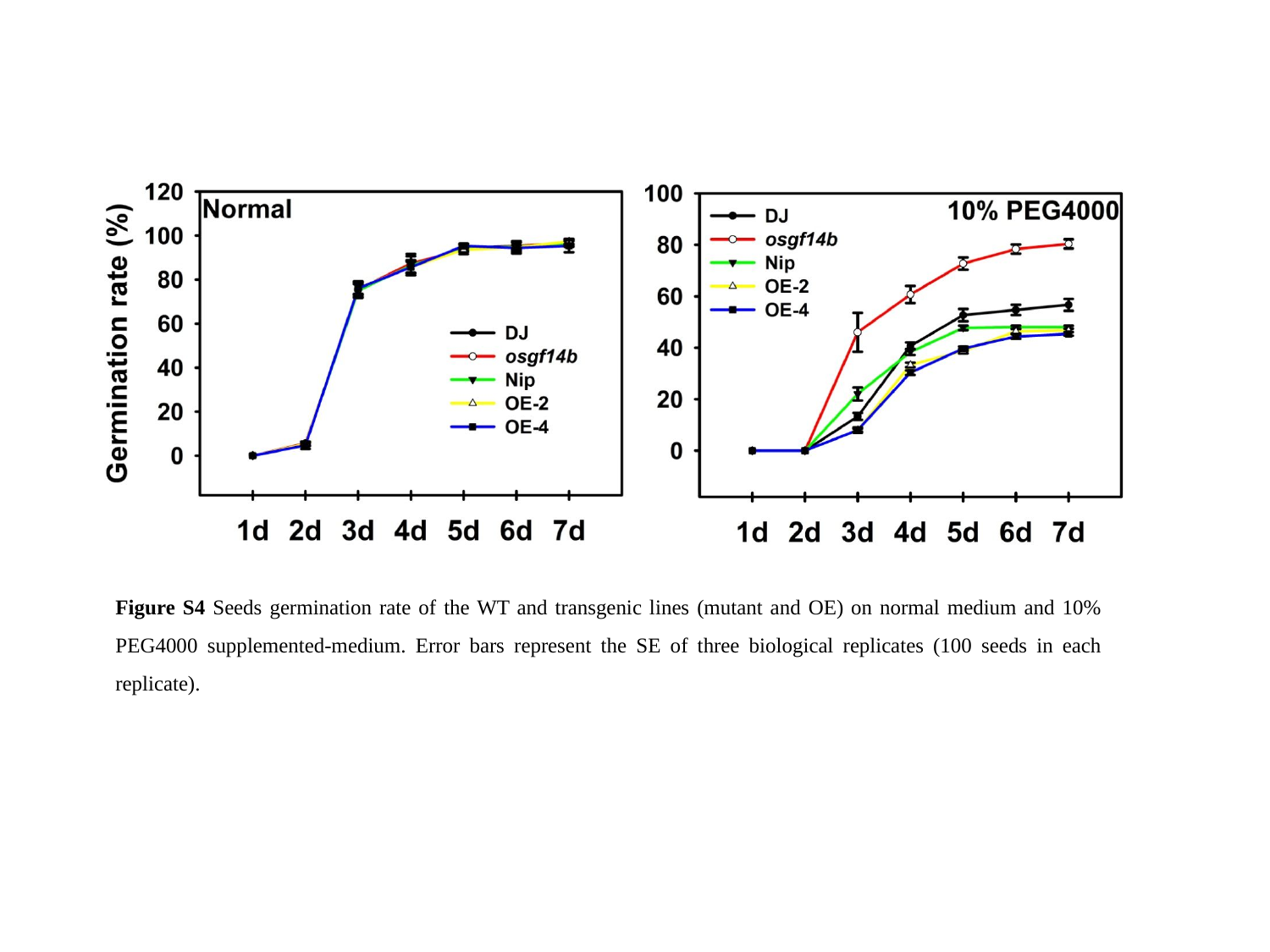

Figure S4 Seeds germination rate of the WT and transgenic lines (mutant and OE) on normal medium and 10% PEG4000 supplemented-medium. Error bars represent the SE of three biological replicates (100 seeds in each replicate).
