# Supplementary material for: Involvement of OsGF14b Adaptation in the Drought Resistance of Rice Plants
Source: Rice (N Y). 2019 Nov 14;12:82. doi: 10.1186/s12284-019-0346-2 (PMC6856252; doi:10.1186/s12284-019-0346-2)
Supplement: Supplementary file 2 — Additional file 2: Table S1. List of primers used in this study (F, forward primer; R, reverse primer; q, quantitative RT-PCR). [file 12284_2019_346_MOESM2_ESM.doc]

**Table S1.** List of primers used in this study (F, forward primer; R, reverse primer; q, quantitative real-time PCR)

| **Primer name** | **Primer sequence (5′→3′)** |
| --- | --- |
| **For qRT-PCR** |  |
| q*OsGF14b*-F | CTTGCAACCTCGCAAAGC |
| q*OsGF14b*-R | GTTATCACGGAGGAGTTGCAT |
| q*Actin1*-F | TGGCATCTCTCAGCACATTCC |
| q*Actin1*-R | TGCACAATGGATGGGCCAGA |
| q*OsNCED4*-F | GATTGCACGGCACCTTCATT |
| q*OsNCED4*-R | CTCTGTAATTTGATTTTTCACTGGCTAAT |
| q*P5CS*-F | TTGGATTGGGTGCTGAGGTTGG |
| q*P5CS*-R | ACGACATCCTTGTCACCATTCACC |
| q*OsbZIP23*-F | GGAGCAGCAAAAGAATGAGG |
| q*OsbZIP23*-R | GGTCTTCAGCTTCACCATCC |
| q*OsLEA3*-F | GCCGTGAATGATTTCCCTTTG |
| q*OsLEA3*-R | CACACCCGTCAGAAATCCTCC |
| q*Rab16c*-F | CCCGGCCAGCACTAAATAAG |
| q*Rab16c*-R | AAACTGCACGTACATCACGACAT |
| **For T-DNA insertion analysis** | |
| mF | AAGATCCGAAACCTGTGCCA |
| mR | TTACTGCCCCGCAATCGT |
| mV | CCGACAGTGGTCCCAAAGAT |
| **For complementation vector construction** | |
| Com-GF14b-F | GC*TCTAGA*ATGTCGGCACAGGCGGA |
| Com-GF14b-R | CG*GGATCC*TTACTGCCCCTCGCTGGAG |
