# Supplementary material for: Involvement of OsGF14b Adaptation in the Drought Resistance of Rice Plants
Source: Rice (N Y). 2019 Nov 14;12:82. doi: 10.1186/s12284-019-0346-2 (PMC6856252; doi:10.1186/s12284-019-0346-2)
Supplement: Supplementary file 3 — Additional file 3. Materials and methods. [file 12284_2019_346_MOESM3_ESM.doc]

**Materials and Methods**

**Plant materials and growth conditions**

The *OsGF14b* mutant line 2D-00086 in the background of the rice variety DongJin (DJ, *Oryza sativa* L. *ssp*. japonica) was obtained from the RISD. Homozygous mutant line was segregated from the heterozygous mutant 2D-00086. Genotyping was performed using the *OsGF14b* genomic primers and the T-DNA border primer (Additional file 2: Table S1). To complement the *osgf14b* mutant, the full coding sequence (CDS) of *OsGF14b* gene was amplified from cDNA of rice leaves using primers Com-GF14b-F and R (Additional file 2: Table S1). The amplified fragment of *OsGF14b* was cloned into the modified vector pCambia1300, which was driven by the 35S promoter. The resulting construct was then introduced into the *Agrobacterium tumefaciens* strain EHA105 and transformed into the *osgf14b* mutant. *OsGF14b*-overexpression lines (OE-2 and OE-4) in the background of the rice variety Nipponbare (Nip, *Oryza sativa* L. *ssp*. japonica) were kindly provided by professor Bin Liu (Rice Research Institute, Guangdong Academy of Agricultural Sciences, Guangzhou, China). All rice plants used in this study were grown in a green house with a 13-h-light (28°C)/11-h-dark (25°C) photoperiod, approximately 70% relative humidity and approximately 200 mmol m^-2^s^-1^ photon densities.

**Drought resistance testing**

To investigate the drought stress resistance of the *osgf14b* mutant, complementation lines and *OsGF14b*-OE lines at the seedling stage, WT and the transgenic rice seeds were sterilized for 30 min with 2% (v/v) sodium hypochlorite (NaClO), followed by thorough rinsing for 30 min with deionized water. Seeds were germinated in darkness at 28 °C for 3 d, and then the uniformly germinated seeds were sown in the same plastic basins or barrels filled with a mixture of sand and soil (1:1). Each plastic basin was planted with about 20 seedlings for DJ, *osgf14b* and two independent complementation lines respectively. After pouring out the water in the basins, the 5.5- to 6.5-leaf stage seedlings were subjected to drought stress treatment with the irrigation was withheld for 12 d. Each plastic barrel was planted with about 10 Nip seedlings and 10 *OsGF14b*-OE seedlings in a half-and-half manner. After pouring out the water in the barrels, the 5.5- to 6.5-leaf stage seedlings of Nip and *OsGF14b-OE* lines were subjected to drought stress treatment with the irrigation was withheld for 8 d. After recovery by re-watering for 7 d, the plants were photographed. Next, the seedlings with newly growing leaf blades were counted as surviving plants and the survival rates were recorded. In addition, before and after drought stress, the leaves from the WT, *osgf14b* and *OsGF14b*-OE lines were collected for detecting the expression of the stress-related genes. To check the expression level of *OsGF14b* under drought treatment, the DJ seedlings at the 4-leaf stage were treated as above, and then the seedling leaves were sampled at each time point. Each stress treatment experiment contained three biological replicates.

**Physiological and Biochemical Analysis**

Before and after drought for 3 d, the fourth complete leaves of the rice plants at 5.5- to 6.5-leaf stage were used for stomatal conductance measurement with a SC-1 leaf porometer (Decagon, USA). Before and after drought stress for 7 d, the leaves from the WT, *osgf14b* and *OsGF14b*-OE lines were collected for measuring the hydrogen peroxide (H_2_O_2_), malondialdehyde (MDA), proline and soluble sugar content. H_2_O_2_ content was detected based on the method of Liu et al., (2018). The MDA, proline and total soluble sugar content in the leaves was determined as previously described with slight modification (Lou et al., 2017).

**PEG, Mannitol and ABA treatment**

To investigate whether OsGF14b functions under osmotic stress, we sowed one hundred surface sterilized seeds for each genotype (WT, mutant and two OE lines) on normal 1/2 MS medium and 10% PEG4000 supplemented-1/2 MS medium. During the germination, we calculated the germination rate at different times (1 d, 2 d, 3 d, 4 d, 5 d, 6 d and 7 d). In addition, after germination on normal 1/2 MS medium for 3 d, the WT and transgenic plants with similar shoot and root length were transplanted to transparent plastic plates with 1/2 MS medium containing 200 mM mannitol or water as a control. After one week of growth, the phenotypes were recorded and shoot length of these seedlings were measured. To test ABA sensitivity, the WT and transgenic lines were germinated on 1/2 MS medium for 3 d. After germination, the seedlings with similar shoot and root length were transplanted to transparent plastic plates with 1/2 MS medium containing 5 μM ABA or water as a control. After one week of growth, the phenotypes were recorded and shoot length of these seedlings were measured.

**RNA Extraction and qRT-PCR Analysis**

The TRIZol reagent (Invitrogen, USA) was used according to the manufacturer's instructions to extract total RNA. Before reverse transcription, total RNA was treated with gDNA Eraser (TaKaRa, Japan) for 5 min at 42°C to degrade possibly contaminated residual genomic DNA. The cDNA templates were synthesized using PrimeScript^TM^ reagent Kit (TaKaRa, Japan) according to the manufacturer’s instructions. Quantitative real-time PCR was performed on an optical 96-well plate with a CFX96 Real-time PCR Detection System (Bio-Rad, USA) using SYBR Premix Ex Taq (TaKaRa, Japan). The PCR thermal cycling protocol was as follows: 95°C for 10 s, followed by 40 cycles at 95°C for 5 s and 60°C for 30 s. The rice *Actin1* gene was used as the internal reference (Zhang et al., 2012), and data analyses with the 2^–ddCt^ method were performed as described (Livak and Schmittgen, 2001). All qRT-PCR experiments described in the research contained three biological replicates, and each replicate had three technical replicates. The primers used in this work are listed in Additional file 2: Table S1.

**References**

Liu J, Sun X, Xu F, Zhang Y, Zhang Q, Miao R, Zhang J, Liang J, Xu W (2018) Suppression of *OsMDHAR4* enhances heat tolerance by mediating H_2_O_2_-induced stomatal closure in rice plants. Rice 11:38

Livak KJ, Schmittgen TD (2001) Analysis of relative gene expression data using real-time quantitative PCR and the 2^-ΔΔCT^ method. Methods 25: 402-408

Lou D, Wang H, Liang G, Yu D (2017) OsSAPK2 Confers Abscisic Acid Sensitivity and Tolerance to Drought Stress in Rice. Front Plant Sci 8:993

Zhang CC, Yuan WY, Zhang QF (2012) *RPL1*, a gene involved in epigenetic processes regulates phenotypic plasticity in rice. Mol Plant 5: 482-493
